# Screening Strategies for Thyroid Disorders in the First and Second Trimester of Pregnancy in China

**DOI:** 10.1371/journal.pone.0099611

**Published:** 2014-06-12

**Authors:** Hong Yang, Minglong Shao, Liangmiao Chen, Qingshou Chen, Lechu Yu, Lingqiao Cai, Zhenzhen Lin, Chi Zhang, Xuemian Lu

**Affiliations:** 1 Department of Endocrinology, The Third Hospital Affiliate to Wenzhou Medical University, Ruian, Zhejiang, China; 2 Ruian Center of the Chinese-American Research Institute for Diabetic Complications, the Third Affiliated Hospital of the Wenzhou Medical University, Ruian, Zhejiang, China; 3 Chinese-American Research Institute for Diabetic Complications, School of Pharmaceutical Sciences, Key Laboratory of Biotechnology Pharmaceutical Engineering, Wenzhou Medical University, Wenzhou, Zhejiang, China; Virgen Macarena University Hospital, School of Medicine, University of Seville, Spain

## Abstract

**Background:**

Thyroid dysfunction during pregnancy is associated with multiple adverse outcomes, but whether all women should be screened for thyroid disorders during pregnancy remains controversial.

**Objective:**

To evaluate the effectiveness of the targeted high risk case-finding approach for identifying women with thyroid dysfunction during the first and second trimesters of pregnancy.

**Methods:**

Levels of thyroid stimulating hormone (TSH), free thyroxine (FT_4_), and thyroid peroxidase antibodies (TPOAb) were measured in 3882 Chinese women during the first and second trimester of pregnancy. All tested women were divided into the high risk or non-high risk groups, based on their history, findings from physical examination, or other clinical features suggestive of a thyroid disorder. Diagnosis of thyroid disorders was made according to the standard trimester-specific reference intervals. The prevalence of thyroid disorders in each group was determined, and the feasibility of a screening approach focusing exclusively on high risk women was evaluated to estimate the ability of finding women with thyroid dysfunction.

**Results:**

The prevalence of overt hypothyroidism or hyperthyroidism in the high risk group was higher than in the non-high risk group during the first trimester (0.8% vs 0, χ^2^ = 7.10, p = 0.008; 1.6% vs 0.2%, χ^2^ = 7.02, p = 0.008, respectively). The prevalence of hypothyroxinemia or TPOAb positivity was significantly higher in the high risk group than in the non-high risk group during the second trimester (1.3% vs 0.5%, χ^2^ = 4.49, p = 0.034; 11.6% vs 8.4%, χ^2^ = 6.396, p = 0.011, respectively). The total prevalence of hypothyroidism or hyperthyroidism and the prevalence of subclinical hypothyroidism or hyperthyroidism were not statistically different between the high risk and non-high risk groups, for either the first or second trimester.

**Conclusion:**

The high risk screening strategy failed to detect the majority of pregnant women with thyroid disorders. Therefore, we recommend universal screening of sTSH, FT_4_, and TPOAb during the first trimester and second trimester of pregnancy.

## Introduction

Thyroid disease during pregnancy is associated with an increase in maternal and fetal risk for a number of adverse effects [1]. Maternal thyroid dysfunction is linked to premature birth, low birth weight, fetal anomaly, fetal death, gestational hypertension, and other pregnancy complications [2], [3]. Hypothyroidism and hypothyroxinemia during the first trimester may impair fetal brain development [4], [5]. Treatment of identified thyroid hormonal abnormalities during pregnancy reduces significant adverse outcomes [6]. Despite the prevalence and risk associated with thyroid disease, controversy regarding universal versus high risk screening strategies remains. Both the Endocrine Society Clinical Practice (ESCP, 2007) [7] and the American Thyroid Association (ATA) guidelines (2011) recommended screening of high risk patients only for thyroid disease in pregnancy [8]. However, according to the Endocrine Society guidelines in 2012, some members advocated for thyroid stimulating hormone (TSH) testing of all women during the first trimester of pregnancy [9].

In this study, we measured TSH, FT4, and TPOAb in pregnant women during the first and second trimester of pregnancy. In order to optimize the identification of thyroid disorders during pregnancy, we compared the effectiveness of high risk screening and universal screening strategies.

## Materials and Methods

### Ethics statements

This study was conducted at the Third Hospital Affiliated to Wenzhou Medical University. The study protocol was approved by the institutional review board of the Third Hospital Affiliated to Wenzhou Medical University. Written informed consent was obtained from all enrolled subjects prior to the study. The individual in this manuscript has given written informed consent (as outlined in PLOS consent form) to publish these case details. Furthermore, we guaranteed the privacy right of each subject was completely observed.

### Subjects

Subjects were selected from women undergoing prenatal examination at the Third Affiliated Hospital of Wenzhou Medical University and Ruian Maternal and Child Care Service Centre between February 2009 and February 2012. Exclusion criteria included women over 28 weeks pregnant and women who lived locally less than five years (the local region is iodine-adequate). A total of 3882 pregnant women enrolled in this study; 1169 in the first trimester of pregnancy and 2713 in the second trimester. The research project design was approved by the ethics committee of the Third Affiliated Hospital of Wenzhou Medical University and all subjects carefully read and signed the informed consent prior to being enrolled in the study. Besides undergoing routine prenatal registration, all the subjects were interviewed by the obstetrician regarding reproductive history (e.g. miscarriages, preterm deliveries, and infertility), personal and family history of thyroid disorders, clinical signs consistent with thyroid disorders, personal history of type 1 diabetes or other autoimmune diseases, history of anti-thyroid drugs use (thyroid hormone or amiodarone treatment), and history of any therapeutic head or neck irradiation. In accordance with the 2011 guidelines of the ATA for the diagnosis and management of thyroid disease during pregnancy and postpartum [8], women were classified as high risk for thyroid disease if their history or physical examination included one or more of the following: thyroid disease and/or thyroid surgery, iodine-131 (I^131^) treatment, family history of thyroid disease, thyroid disease symptoms, thyroid swelling, positive for thyroid autoantibodies, type 1 diabetes or other autoimmune diseases, infertility, spontaneous abortion, preterm birth, amiodarone or lithium treatment, head and neck irradiation, recent exposure to iodine contrast agent, age greater than 30, and body mass index greater than 40 kg/m^2^.

### Methods of sampling and laboratory testing

Following an overnight fast, blood samples were acquired in the morning from each subject. Serum was isolated by centrifugation and stored at −80°C until testing. The concentration of serum TSH and FT_4_ were detected by a electrochemiluminescence immunoassay diagnostic kit (Beckman Coulter, Suzhou, China). TPOAb levels were detected using an electrochemiluminescence immunoassay diagnostic kit (Roche Diagnositics Ltd., Basel, Switzerland) and analyzed on an E601 module immunology analyzer (Roche Diagnostics Ltd.). Intra-and inter-assay variability had coefficient of variation (CV) values less than 10%.

### Trimester-specific reference intervals TSH and FT_4_ and diagnostic criteria used to classify study subjects

According to the standard of the National Academy of Clinical Biochemistry (NACB) [10], first trimester, TSH 0.09–3.47 mIU/L and FT4 7.74–15.80 pmol/L; second trimester, TSH 0.20–3.81 mIU/L and FT4 5.55–12.56 pmol/L. Overt and subclinical hypothyroidism were defined as increased TSH and decreased or normal FT4 levels. Overt and subclinical hyperthyroidism were defined as decreased TSH, and increased FT4 or normal FT4 levels, respectively. Hypothyroxinemia was defined as normal TSH, decreased FT4. A TPOAb concentration ≥34 IU/ml was considered abnormal.

### Statistical analysis

Statistical analysis was performed using SPSS software (version 19.0; SPSS Inc., Chicago, IL, USA). Statistical comparisons were made using the chi-squared test, and if significant, variables were further compared by multivariate logistic regression analysis. All data are expressed as mean ± standard deviation or percentages, where P value≤0.05 was considered statistically significant.

## Results

### Characteristics and history of pregnant women

Demographic information of the patients enrolled in the study is shown in Table 1. The average age of the 1169 women in the first trimester was 26.6±3.5 years (191 women were over 30 years, 16.3%), and the median duration of pregnancy was 9 weeks (range 3–12 weeks). In the high risk group, there were 257 women (22.0%), and 188 tested positive for TPOAb (10.1%).

**Table 1:** Demographic characteristics of the pregnant women in this study (n = 3882).

Demographic characteristics	First trimester	Second trimester
Number of cases, n	1169	2713
Mean maternal age in years, x±s	26.6±3.5	27.2±3.7^**^
Age older 30	191 (16.3%)	605 (22.3%)^**^
Body index greater than 40 kg/m^2^	0	0
Median (range) gestational age at screening in weeks	9 (3–12)	23 (13–27)
Number of previous pregnancies, n (%)		
One	369 (31.6%)	847 (31.2%)
Two	137 (11.7%)	390 (14.4%)
Three or more	119 (10.2%)	404(14.9%)
History of miscarriages, n (%)	67 (6.7%)	114 (4.2%)^△^
History of premature birth, n (%)	5 (0.4%)	4 (0.1%)
History of infertility, n (%)	0	4 (0.1%)
History of smoking, n (%)	2 (0.2%)	10 (0.4%)
Personal history of thyroid disease, n (%)	7 (0.6%)	16 (0.4%)
Hyperthyroidism	1 (0.1%)	4 (0.1%)
Hypothyroidism	2 (0.2%)	4 (0.1%)
Goiter/nodule	1 (0.1%)	4 (0.1%)
Other thyroid disease	3 (0.3%)	4 (0.1%)
Family history of thyroid disease, n (%)	6 (0.5%)	11 (0.4%)
History of head or neck irradiation or treatment with amiodarone or lithium, n (%)	0	0
History of exposure to iodinated contrast agents, n (%)	0	0
Type 1 diabetes/autoimmune disease	2 (0.2%)	4 (0.1%)
Positive TPOAb	118 (10.1%)	250 (9.2%)
High risk group	257 (22%)	708 (26.1%)^*^

Notes: Data are presented as mean±SD, first trimester vs. second trimester ^**^ *p*<0.000, ^*^ *p*<0.01, ^△^ *p*<0.05.

The average age of the 2713 women in the second trimester was 27.17±3.74 years (605 women were over 30 years, 22.3%), and the median duration of pregnancy was 23 weeks (13–27 weeks). In the high risk group, there were 708 women (26.1%), and 250 tested positive for TPOAb (9.2%).

Between women in the first and second trimesters of pregnancy, there was no significant difference in the number of previous pregnancies, smoking habits, history of preterm delivery, history of infertility treatment, personal history of thyroid disorders, family history of thyroid disorders, type 1 diabetes or other autoimmune diseases, or TPOAb positivity. However, the second trimester group was significantly older than the first trimester group (p<0.000). In addition, there were more patients with high risk factors in the second trimester group than in the first trimester group (p<0.01). Regarding history of miscarriage, the rate of miscarriage was significantly lower in the second trimester group than the first trimester group (p<0.05).

### Comparison between universal and high risk screening of thyroid disorders in the first trimester of pregnancy

The prevalence of thyroid disorders in the first trimester of pregnancy is shown in table 2. Of the 1169 first trimester women evaluated, two hundred fifty-seven women were categorized as high risk, and 912 were categorized as non- high risk. Sixty-three women (5.4%) had thyroid dysfunction, which included 12 (1.0%) with hyperthyroidism, 51 (4.4%) with hypothyroidism; fifteen women (1.3%) had low level of FT_4_ and 118 (10.1%) had positive TPOAb test.

**Table 2:** Prevalence of thyroid disorders in the first trimester (n, %).

	High risk group n = 257	Non-high risk group n = 912	Total n = 1169	High risk group vs Non-high risk group	
				?^2^	p
Hypothyroidism	9 (3.5)	42 (4.6)	51(4.4)	0.58	0.444
Overt	2 (0.8)	0	2(0.2)	7.10	0.008
Subclinical	7 (2.7)	42 (4.6)	49 (4.2)	1.77	0.219
Hyperthyroidism	5 (1.9)	7(0.8)	12(1.0)	2.74	0.098
Overt	4 (1.6)	2(0.2)	6(0.5)	7.02	0.008
Subclinical	1 (0.3)	5(0.6)	6(0.5)	0.10	0.752
Hypothyroxinemia	4(1.6)	11(1.2)	15(1.3)	0.19	0.659
TPOAb positive	30(11.7)	88(9.6)	118(10.1)	0.91	0.341

Of the 51 women with elevated TSH, two (0.2%) had overt hypothyroidism and 49 (4.2%) had subclinical hypothyroidism. In the high risk group, nine women (3.5%) had hypothyroidism, two (0.8%) had overt hypothyroidism, and seven (2.7%) had subclinical hypothyroidism. Therefore, in the non-high risk group, 42 women (4.6%) had subclinical hypothyroidism, accounting for 82.4% of all cases of hypothyroidism, and none had overt hypothyroidism. The prevalence of overt hypothyroidism in the high risk group was significantly higher than in the non-high risk group (0.8% vs 0, χ^2^ = 7.10, p = 0.008), but there was no significant difference between high risk and non-high risk groups in the overall prevalence of hypothyroidism or subclinical hypothyroidism (3.5% vs 4.6%, χ^2^ = 0.58, p = 0.444) or subclinical hypothyroidism (2.7% vs 4.6%, χ^2^ = 1.77, p = 0.219).

Twelve women (1.0%) had low TSH, including six (0.5%) with overt and six (0.5%) with subclinical hyperthyroidism. Within the high risk group, five (1.9%) women had hyperthyroidism, including four (1.6%) with overt and one (0.3%) with subclinical hyperthyroidism. In the non-high risk group, seven (0.8%) women had hyperthyroidism, accounting for 58.3% of hyperthyroidism overall, where two(0.2%) had overt hyperthyroidism and five (0.6%) had subclinical hyperthyroidism. The overall prevalence of hyperthyroidism or subclinical hyperthyroidism in both groups was not significantly different (1.9% vs 0.8%, χ^2^ = 2.74, p = 0.098; 0.3% vs 0.6%, χ^2^ = 0.10, p = 0.752, respectively), but the prevalence of overt hyperthyroidism in the high risk group was significantly higher than in the non-high risk group [1.6% vs 0.2%, χ^2^ = 7.02, odds ratio, OR = 7.19,95% CI (1.31-39.50), p = 0.008].

Overall, 15 women (1.3%) had normal TSH and decreased FT_4_ and were diagnosed with hypothyroxinemia, including four women (1.6%) in the high risk group and 11 women (1.2%) in the non-high risk group (accounting for 73.3% of all cases of hypothyroxinemia). The prevalence of low thyroid hormone was almost the same in both groups (1.6% vs 1.2%, χ^2^ = 0.19 p = 0.659).

Regarding the TPOAb test, 118 (10.1%) women tested positive for TPOAb, where 30 (11.7%) were categorized as high risk and 88 (9.6%) were categorized as non- high risk (accounting for 74.6% of all cases of positive TPOAb test). There was no significant difference between the prevalence of a positive TPOAb test in either of the groups (11.7% vs 9.6%, χ^2^ = 0.91, p = 0.341). Of the 118 pregnant women who tested positive for TPOAb, 13 (11.0%) were diagnosed with thyroid disorders, including ten (8.5%) women with subclinical hypothyroidism, one (0.8%) with overt hypothyroidism, and two (1.7%) with hypothyroxinemia. However, among euthyroid pregnant women, 9.0% tested positive for TPOAb.

The risk factors of thyroid disorders in the first trimester of pregnancy are shown in table 3.Women with a history of thyroid disease were more likely than women without to have maternal hypothyroidism (42.9% vs 4.1%, χ^2^ = 25.01, p = 0.002). In addition, the prevalence of hypothyroidism in TPOAb-positive women was significantly higher than TPOAb-negative women (9.3% vs 3.8%, χ^2^ = 7.73, p = 0.014). Consistent with previous findings, logistic multiple regression analysis in these patients showed that history of thyroid disease (OR = 17.13, p = 0.001) and a positive TPOAb test (OR = 2.34, p = 0.020) were risk factors for hypothyroidism during pregnancy. The prevalence of hyperthyroidism in women over the age of 30 was significantly higher than women less than 30 years. Logistic multiple regression analysis revealed that women over 30 years were at greater risk than women younger than 30 for hyperthyroidism (OR = 10.93, p = 0.006).

**Table 3:** Risk factors of thyroid disorders in the first trimester (n, %).

Characteristics	n	Low TSH			High TSH			Normal TSH
		High FT4	Normal FT4	Total	Low FT4	Normal FT4	Total	Low FT4
TPOAb positive	118	0	0	0	1 (0.8)	10 (8.5)	11 (9.3)	2 (1.7)
History of thyroid disease	7	0	0	0	1(14.3)	2 (28.6)	3 (42.9)	0
Family history of thyroid disease	6	0	0	0	0	0	0	0
Type 1 diabetes/autoimmune disease	2	0	0	0	0	0	0	0
History of miscarriages	67	0	0	0	0	1(1.5%)	1(1.5%)	0
History of premature birth	5	0	0	0	1(20%)	0	1(20%)	0
History of infertility	0	0	0	0	0	0	0	0
Age older than 30	191	4(2.1)	1(0.5)	5(2.6)	2 (1.0)	5 (2.6)	7 (3.7)	4 (2.1)

### Comparison between universal screening and high risk screening of thyroid disorders in the second trimester of pregnancy

The prevalence of thyroid disorders in the second trimester of pregnancy is shown in table 4. Of the 2713 second trimester women evaluated, 708 women were categorized as high risk, and 2005 were categorized as non-high risk. Seventy-seven women (2.8%) had thyroid dysfunction, which included 19 women (0.7%) with hyperthyroidism, 58 (2.1%) with hypothyroidism; 19 women (0.7%) had low level of FT_4_ and 250 (9.2%) had positive TPOAb test.

**Table 4:** Prevalence of thyroid disorders in the second trimester (n, %).

	High risk group n = 708	Non-high risk group n = 2005	Total n = 2713	High risk group vs Non-high risk group	
				?^2^	*P* value
Hypothyroidism	16 (2.3)	42 (2.1)	58 (2.1)	0.07	0.764
Overt	1 (0.1)	3 (0.1)	4(0.1)	0.00	1.000
Subclinical	15 (2.1)	39 (1.9)	54 (2.0)	0.08	0.756
Hyperthyroidism	3 (0.4)	16 (0.8)	19 (0.7)	1.05	0.305
Overt	2 (0.3)	11 (0.5)	13 (0.5)	0.78	0.534
Subclinical	1 (0.1)	5 (0.3)	6 (0.2)	0.28	0.599
Hypothyroxinemia	9 (1.3)	10 (0.5)	19 (0.7)	4.49	0.034
TPOAb positive	82 (11.6)	168 (8.4)	250 (9.2)	6.396	0.011

Of the 58 women with elevated TSH, four (0.1%) had overt hypothyroidism and 54 (2.0%) had subclinical hypothyroidism. In the high risk group, 16 women (2.3%) had hypothyroidism, including one (0.1%) with overt hypothyroidism and 15 (2.1%) with subclinical hypothyroidism. From the non-high risk group, 42 women (2.1%) had hypothyroidism, accounting for 72.4% of all cases of hypothyroidism, including three (0.1%) with overt hypothyroidism and 39 (1.9%) with subclinical hypothyroidism. There was no significant difference between the high risk and non-risk groups regarding prevalence of hypothyroidism (2.3% vs 2.1%, χ^2^ = 0.07, p = 0.764) and subclinical hypothyroidism (2.1% vs 1.9%, χ^2^ = 0.08, p = 0.756). The prevalence of overt hypothyroidism was almost the same in both groups (0.1% vs 0.1%, χ^2^ = 0.00, p = 1.000).

Nineteen women (0.7%) were diagnosed with hyperthyroidism, including 13 (0.5%) with overt and six (0.2%) with subclinical hyperthyroidism. In the high risk group, three (0.4%) women had hyperthyroidism, including two (0.3%) with overt and one (0.1%) with subclinical hyperthyroidism. In the non-high risk group, 16 (0.8%) women had hyperthyroidism, accounting for 84.2% of hyperthyroidism overall, with 11 (0.5%) overt and five (0.3%) subclinical cases of hyperthyroidism. The prevalence of hyperthyroidism in both groups was not significantly different (0.4% vs. 0.8%, χ^2^ = 1.05, p = 0.305). The prevalence of overt hyperthyroidism or subclinical hyperthyroidism in both groups was not significantly different (0.3% vs. 0.5%, χ^2^ = 0.78, p = 0.534; 0.1% vs. 0.3%, χ^2^ = 0.28, p = 0.599, respectively).

Overall, 19 (0.7%) women had normal TSH and decreased FT_4_ and were diagnosed with hypothyroxinemia, including nine women (1.3%) in the high risk group and ten women (0.5%) in the non-high risk group (accounting for 52.6% of hypothyroxinemia overall). The prevalence of hypothyroxinemia in the high risk group was significantly higher than in the non-high risk (1.3% vs 0.5%, χ^2^ = 4.49, p = 0.034).

Regarding the TPOAb test, 250 women tested positive for TPOAb, where 82 (11.6%) women were categorized in the high risk group and 168 (8.4%) were categorized in the non-high risk group (accounting for 67.2% of all cases of positive TPOAb test). The prevalence of a positive TPOAb test in the high risk group was significantly higher than the non-high risk group (11.6% vs 8.4%, χ^2^ = 6.396, p = 0.011). Of the 250 pregnant women who tested positive for TPOAb, 16 were diagnosed with thyroid disorders, including 10 with subclinical hypothyroidism, one with overt hypothyroidism, two with isolated hypothyroxinemia, and three with hyperthyroidism. Among euthyroid pregnant women, 8.9% were positive for TPOAb.

The risk factors of thyroid disorders in the second trimester of pregnancy are shown in table 5. The prevalence of hypothyroidism during pregnancy in women who had a personal history of thyroid disorders, positive TPOAb test, or history of preterm delivery was significantly higher than women in the null corresponding groups (35.3% vs 1.9%, p = 0.000; 4.8% vs 1.8%, p = 0.002; 25.0% vs 2.1%, p = 0.002, respectively). Logistic multiple regression analysis showed that a personal history of thyroid disorders (OR = 25.29, p = 0.000), TPOAb positivity (OR = 2.29, p = 0.019), and history of preterm delivery (OR = 21.91, p = 0.009) were risk factors for the presence of hypothyroidism during pregnancy. The prevalence of isolated hypothyroxinemia in women over 30 years was significantly greater than women less than 30 years. Logistic multiple regression analysis showed that women over 30 years (OR = 3.16, p = 0.013) were at greater risk for hypothyroxinemia.

**Table 5:** Risk factors of thyroid disorders in the second trimester (n %).

Characteristics	n	Low TSH			High TSH			Normal TSH
		High FT4	Normal FT4	Total	Low FT4	Normal FT4	Total	Low FT4
TPOAb positive	250	3 (1.2)	0	3 (1.2)	1 (0.8)	10 (8.5)	11 (9.3)	2 (1.7)
History of thyroid disease	16	0	0	0	1 (6.6)	5 (31.2)	6 (37.5)	0
Family history of thyroid disease	11	0	0	0	0	0	0	0
Type1 diabetes/autoimmune disease	4	0	0	0	0	0	0	0
History of miscarriages	114	0	0	0	0	2 (1.8)	2 (1.8)	1 (0.9)
History of premature birth	4	0	0	0	1(25%)	1 (25%)	1 (20%)	0
History of infertility	4	0	0	0	0	0	0	0
Age older than 30	605	2 (0.3)	1 (0.2)	3(0.5	1 (0.2)	9 (1.5)	10 (1.7)	9 (1.5)

## Discussion

Overt hypothyroidism is regarded as a major risk factor for complications of pregnancy and neurocognitive deficits in the developing fetus [11], [12]. In recent years, there is mounting evidence demonstrating that even subclinical hypothyroidism can produce deficits similar to overt hypothyroidism. In children (7–9 years) born to women hypothyroid during pregnancy, there is a seven point deficit in intelligence quotient (IQ) score and delays in motor, language, and attention [4]. In addition, the children of mothers with hypothyroxinemia during pregnancy exhibit neurodevelopmental delay [13]. It was reported in 28 out of 31 similar studies that subclinical hypothyroidism increased the risk of adverse outcomes of pregnancy [14]. Treatment of hypothyroidism or hyperthyroidism in screened low risk patients was associated with a decrease in the rate of adverse outcomes [6]. Uncontrolled thyrotoxicosis during pregnancy was associated with miscarriage, pregnancy-induced hypertension, premature birth, low birth weight, fetal growth restriction, still birth, thyroid crisis, and congestive heart failure [15]–[17]. Therefore, early diagnosis of thyroid dysfunction during pregnancy and onset of rational therapy can alleviate the adverse outcomes of pregnancy.

There is ongoing, widespread controversy regarding the necessity of universal screening of thyroid function in pregnant women. The Endocrine Society Clinical Practice guideline [7] recommended a case-finding screening strategy in 2007. However, the effectiveness of this screening strategy has been questioned. Vaidya et al. [18] found that by only screening high risk women, between 30 and 69% of women with thyroid dysfunction during pregnancy would go undiagnosed. Consistent with this, Wang et al. [19] reported that a case-finding screening strategy would miss 81.6% of pregnant women with hypothyroidism and 80.4% of pregnant women with hyperthyroidism. The new guidelines of the American Thyroid Association recommend only a case-finding screening strategy targeted at high-risk women [8]. A change from the previous guidelines from 2007 from The Endocrine Society was the addition of the age over 30 years risk factor.The Endocrine Society guidelines were updated in 2012, and some members recommended screening all pregnant women for serum TSH abnormalities by the ninth week or at the time of their first visit, whereas other members were neither for nor against universal screening [9].

In this study, we analyzed the effectiveness of the new ATA guidelines regarding screening pregnant women for thyroid disorders. Although screening for thyroid disorders in the early pregnancy is recommended [7], [8], often times women in underdeveloped areas fail to seek prenatal treatment until the second trimester. In our study, a large proportion of women who were receiving prenatal care for the first time were already in the second trimester of pregnancy. Thyroid disease if left untreated will continue throughout the entire pregnancy. Thus, screening for thyroid disorders in the second trimester is also important. Since the ATA supports the use of assay-specific, trimester specific reference intervals to define thyroid dysfunction during pregnancy [8], according to the standard of the National Academy of Clinical Biochemistry (NACB) [10], we formulated independent trimester-specific normal reference intervals for thyroid function, and all pregnant women were diagnosed according to the reference values. In our study, the upper limit of TSH for diagnosis of hypothyroidism was 3.47 mU/L in the first trimester and 3.81 mlU/L in the second trimester, which was similar to the cutoff used by Lazarus et al. [20]. According to our results, 100% (2 of 2) of women with overt hypothyroidism and 66.7% (4 of 6) of women with overt hyperthyroidism would have been identified by high risk screening strategy in the first trimester. History of thyroid disease and TPOAb positivity were risk factors for hypothyroidism during pregnancy and age 30 or older was a risk factor for hyperthyroidism or hypothyroxinemia. However, if only high-risk pregnant women were screened for thyroid disease, as the guidelines recommend, 82.4% of women with subclinical hypothyroidism, 58.3% of women with hyperthyroidism (28.6% overt hyperthyroidism and 71.4% subclinical hyperthyroidism), and 73.3% of women with isolated hypothyroxinemia would have been missed in the first trimester; and 72.4% of women of hypothyroidism (7.1% overt hypothyroidism and 92.9% subclinical hypothyroidism), 84.2% of women of hyperthyroidism (68.7% overt hyperthyroidism and 31.3% subclinical hyperthyroidism), and 52.6% of women of isolated hypothyroxinemia would have been missed in the second trimester. Our results were similar to those reported by Wang et al. [19]. Negro et al. [6] also found that case finding fails to detect the majority of pregnant women with thyroid disease. Recently, Potlukova et al. [21] reported that addition of age 30 or older increased the proportion of women identified in a case-finding screening strategy from 55.3 to 85.6%. However, the authors indicated that this high proportion was valid for a relatively older population of pregnant women, with an average age of 31. If there was a lower average age, the proportion of hypothyroid women identified by the screening would decrease. In our study, the average age of the women in the first trimester was 26.6±3.5 years (only 16.3% were over 30 years); and the average age of the women in the second trimester was 27.17±3.74 years (22.3% of women were over 30). Therefore, the efficiency of the case-finding screening strategy may have been decreased in our study because the average age of the population was less than the previous study.

Targeted screening of high risk pregnant women appears to only work for those women with overt hypothyroidism, rather than subclinical hypothyroidism; a possible reason for this finding from our current study is that the women making up the study population were classified as high risk for thyroid disorders based upon their history, findings from physical examination, or findings of other clinical features suggestive of a thyroid disorder. Nevertheless, the majority of these women were found to have overt hypothyroidism.

In particular, we found that 9.0% and 8.9% of euthyroid pregnant women had a positive TPOAb test in the first or second trimester, respectively. Several other studies have demonstrated that a positive TPOAb test increased the risk of miscarriage, premature birth and other pregnancy complications [22], [23]. In euthyroid pregnant women with positive TPOAb, TSH levels gradually increased during the course of pregnancy, and 19% of women in that subgroup had elevated TSH at the time of delivery [24]. Another study examining euthyroid women with positive TPOAb test found that 16% of these women in that subgroup had TSH>4 mIU/L at delivery [25]. Therefore, it is necessary to regularly monitor thyroid function in TPOAb positive women in order to maintain optimal maternal and fetal health. In our study, if only the high-risk pregnant women were tested, 74.6% and 67.2% of women positive for TPOAb would have been missed in the first or second trimester, respectively.

Regarding the cost-effectiveness of screening strategies, Dosiou et al. [26] reported that universal screening of autoimmune thyroid disease in the first trimester was cost-effective, not only compared with no screening but also compared with screening of high-risk women. Others have proposed if L-T4 therapy was efficacious in preventing neurodevelopmental delay and IQ deficits, universal screening for subclinical hypothyroidism in pregnancy would be cost-effective long-term [27], given that testing is widely available, easy, and relatively inexpensive. Therefore, universal screening for TSH, FT_4_, and TPOAb is more cost-effective than screening the high risk group alone.

The limitation of this study was that all participants were from the coastal region of southern China, and an obstetrician, not a physician, obtained information regarding risk factors. Thus, the data may be biased. However, as previously mentioned, a number of studies, including a randomized controlled trial study [6], have demonstrated that the efficiency of a high risk screening strategy is lower than a universal screening strategy.

## Conclusions

In summary, our study confirms that a high risk screening strategy where only women in the high risk group are tested would miss the majority of thyroid disorders in pregnant women. We believe universal screening of sTSH, FT_4_, and TPOAb is essential during the first trimester and second trimester of pregnancy. Taken together, we support implementation of a universal screening strategy for thyroid disorders in pregnant women
